# A cross-sectional analysis of factors associated with the teachable moment concept and health behaviors during pregnancy

**DOI:** 10.1186/s12884-024-06348-8

**Published:** 2024-02-20

**Authors:** Linda M. Uzan, Michelle Brust, Joyce M. Molenaar, Eva Leistra, Klarke Boor, Jessica C. Kiefte-de Jong

**Affiliations:** 1 Health Campus The Hague/Department of Public Health and Primary Care, Leiden University Medical Center, The Hague, The Netherlands; 2 National Institute for Public Health and the Environment (RIVM), Centre for Nutrition, Prevention and Health Services, Department of Quality of Care and Health Economics, Bilthoven, The Netherlands; 3 Department of Health Sciences, Faculty of Science, Amsterdam Public Health Research Institute, Vrije Universiteit Amsterdam, Amsterdam, The Netherlands; 4 Department of Gynecology, Leiden University Medical Centre, Leiden, The Netherlands

**Keywords:** Teachable moment, Pregnancy, Health, Behavior change, Lifestyle, Prevention

## Abstract

**Background:**

Pregnancy is often associated with a change in health behaviors, leading some to suggest that pregnancy could be a teachable moment for lifestyle change. However, the prevalence and underlying mechanism of this phenomenon is not well understood. The aim of this study is to explore the prevalence of a teachable moment during pregnancy, the psychosocial factors that are associated with experiencing such a moment, and its association with actual health behaviors.

**Methods:**

In this cross-sectional study, 343 pregnant Dutch women completed an online questionnaire. Participants reported on their intentions to change lifestyle due to pregnancy, their current health behaviors, and several psychosocial factors that were assumed to be linked to perceiving a teachable moment during pregnancy: perceived risk, affective impact, changed self-concept, and social support. Multivariable linear and logistic regression were applied to the data analysis.

**Results:**

Results demonstrate that 56% of the women experienced a teachable moment based on intentions to change their health behavior. Multivariate regression analyses revealed that changed self-concept (β = 0.21; CI = 0.11–0.31), positive affect (positive β = 0.28; CI = 0.21–0.48), and negative affect (β = 0.12; CI = 0.00-0.15) were associated with higher intentions to change health behavior. Conversely, more perceived risk was associated with lower intentions to change health behavior (β=-0.29; CI = 0.31 − 0.13). Multivariate regression analyses showed a positive association between intentions to change health behavior and diet quality (β = 0.11; CI = 0.82–1.64) and physical activity (OR = 2.88; CI = 1.66-5.00).

**Conclusions:**

This study suggests that pregnancy may be experienced as a teachable moment, therefore providing an important window of opportunity for healthcare professionals to efficiently improve health behaviors and health in pregnant women and their children. Results suggest that healthcare professionals should link communication about pregnancy-related health behaviors to a pregnant women’s change in identity, affective impact (predominantly positive affective impact) and risk perception to stimulate the motivation to change healthy behavior positively.

**Supplementary Information:**

The online version contains supplementary material available at 10.1186/s12884-024-06348-8.

## Introduction

During pregnancy, maternal lifestyle impacts the development and long-term health and wellbeing of the baby [1]. This is explained by the Developmental Origins of Health and Disease (DOHaD) theory. This theory states that exposure to stress, environmental toxins, undernutrition or overnutrition during critical periods of development can have long term effects on offspring’s health and wellbeing [2]. Health behaviors that may adversely affect fetal development or health later in life are: inadequate nutrition (including folic acid and vitamin D supplementation) [3], inadequate physical activity [4, 5], smoking [6, 7] and alcohol use [8, 9]. Consequently, the association between maternal health behaviors and the baby’s health may make future mothers more motivated to adapt these health behaviors during their pregnancy in order to protect the baby’s (future) health [10, 11].

Behavioral theories underpinning individual behavior change, such as the Health Belief Model, accentuate the importance of ‘cues’ in promoting motivation for behavior change [12]. A particular type of cue, referred to as a ‘teachable moment’ (TM), can be defined as ‘a naturally occurring life event or circumstance that could motivate individuals to spontaneously adopt risk-reducing health behaviors’ [10]. During these TM’s, individuals are more receptive to lifestyle advice and more inclined to change health behavior [10, 13]. In practice TM’s are scarcely recognized and utilized [14].

Pregnancy has the potential to be a prime TM. Pregnancy is a major life event that represents the transition to motherhood, often changing people’s identities, lives and the outlook thereof [15, 16]. These changes can accommodate spontaneous behavior change, which is confirmed by a prospective study showing spontaneous reduction of smoking and alcohol consumption during pregnancy of 44% and 80%, respectively [17]. Furthermore, pregnancy involves recurrent contact with health professionals, creating an important window of opportunity for advice and primes regarding health behavior change [18, 19].

Despite their potential implications for health promotion, TM’s have received limited attention in research and theoretical development [11]. Whether or not a life event will instigate health behavior change depends on its impact on several psychosocial factors. McBride et al. [10] were first to develop a conceptual framework of TM’s. According to their initial heuristic model, a life event should (1) increase *risk perception* (2) induce a strong emotional or *affective impact*, and (3) lead to a considerable change in or challenge to a person’s *self-concept* [10]. This TM model has been empirically tested and proven effective in explaining smoking cessation behavior of patients and close relatives receiving a diagnosis of cancer [20, 21]. Moreover, there is evidence that *social support* is also associated with changing health behaviors in pregnant women [22–24].

Since Phelan [25] suggested pregnancy to possibly be a TM, several studies have addressed health behavior change during pregnancy [19, 25–29]. However, only few applied the McBride TM model during pregnancy and mostly focused on specific risk groups. For example, Okely et al. [30] applied the TM model in a study on determinants of effective gestational diabetes mellitus self-management. Risk perception was found to be associated with increased treatment adherence, however, the strongest association was found for social support.

**Fig. 1:**
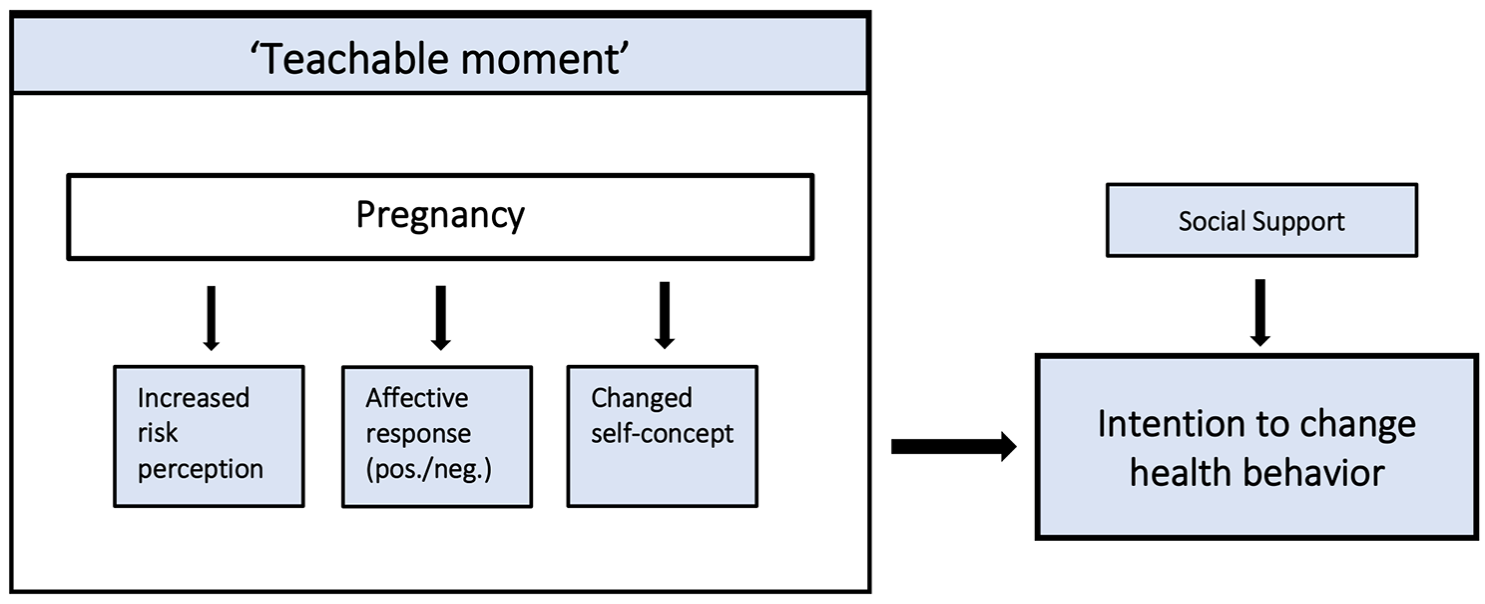
Potential conceptual framework adapted from McBride et al. [10]. Displays the conceptual framework that will be explored schematically. We hypothesize that pregnancy can increase intentions to change health behavior when it leads to; (1) increased risk perception (2) positive or negative affective response, (3) changed self-concept, based on McBride’s framework [10]. Social support is the fourth factor in this conceptual framework, which was added to the initial framework as this was considered to be a relevant factor in previous TM literature in pregnant women [22, 24, 31]

It remains unclear if uncomplicated pregnancy can also be a TM and if so, which psychosocial factors are associated with experiencing such a TM during pregnancy. The first aim of this study is therefore to investigate in what proportion of pregnant women an intention to change lifestyle is provoked by pregnancy. The second aim is to investigate whether risk perception, affective impact, and changed self-concept, based on the conceptual framework described by McBride et al. [10], and social support, are associated with lifestyle change intentions among pregnant women (Fig. 1). The third aim is to understand the association between lifestyle change intentions and current self-reported health behaviors. More understanding about the potential of pregnancy as a TM, the psychosocial factors that are associated with this, and the type of behaviors that are more prone to change, can aid the development of tailored lifestyle interventions around pregnancy.

## Methods

### Study design, participant recruitment and study procedures

Recruitment and data collection for this cross-sectional study took place between May and July, 2021. Participants were recruited via an online or on-paper flyer which contained information about the study and a link and QR code. Women could access the online questionnaire by clicking the link or scanning the QR code. The flyers were placed at 14 midwifery practices in and around Leiden, the Bronovo hospital, the Leiden University Medical Centre (LUMC), a general practitioner, and a pelvic physiotherapist. Additionally, a patient organization supporting pregnant women helped recruiting participants. Finally, online flyers were distributed on Facebook, Linked-In, and relevant fora (“24 baby”, “Brabbels”, “Wij ouders”, “Babybytes” and “Ouders van nu”). Gift vouchers of a Dutch baby store (4 × 25 euros) were raffled among the participants as an incentive to participate. The online survey software program Qualtrics (www.Qualtrics.com) was used. Inclusion criteria were being 18 years or older, being currently pregnant which is already confirmed by an ultrasound, and being able to read Dutch. Data collection was anonymous. After providing informed consent online, participants were eligible to start the questionnaire. A previous cross-sectional study of Okely et al. [30] among pregnant women investigated psychosocial factors associated with concordance with advice on gestational diabetes regulation (i.e. regarding diet, glucose monitoring, and medication intake) used a sample size of *n* = 80 to achieve an effect size of 0.18 (80% power with α = 0.05). The effect size for the present study was hypothesized at 0.09 based on the assumption that the expected effect would be smaller due to the absence of an illness. A power calculation showed that we need 184 participants to achieve this (80% power with α = 0.05 and estimated effect size Cohen’s ƒ^2^ of 0.09). Over a 7-week period, 438 women started the questionnaire, off which 347 finished, resulting in a survey completion rate of 79%. After checking the data for duplicates based on IP addresses, the final sample consisted of 343 participants. The study was conducted in accordance with the Declaration of Helsinki. The Medical Ethics Committee of the LUMC approved and registered this addendum on June 13th 2021 (METC-nr 18–112). All participants provided digital informed consent.

### Study measures

Below, we will specify which scales were used in the online questionnaire. All scales are provided in Additional file 1.

#### Psychosocial factors

The Cardiac Teachable Moment (CardiacTM) scale (alpha = 0.88) was used as a basis for the assessment of the psychosocial factors [32]. This validated scale was developed based on McBride’s conceptual framework and assesses risk perception, affective impact, and changed self-concept due to an acute cardiac event. In the current study, the first psychosocial factor “*Increased risk perception”* was measured using two subscales. *“Perceived risk lifestyle”* was measured by a subscale of the CardiacTM questionnaire, adjusted to the event of pregnancy (e.g. “My current lifestyle is bad for my heart” was reframed into “My current lifestyle is bad for me and my baby”). The final subscale consisted of five items that could be answered on a 7-point Likert scale. Then, “*Perceived risk pregnancy”* was measured by an adapted version of Heaman and Gupto’s Perception of Pregnancy Risk Questionnaire [33]. Four of the original nine items were used in our current study, of which two items that measured risk for the baby (low birthweight and being born prematurely) and two that measured risk for the mother (gestational diabetes and high blood pressure or (pre)eclampsia). These four complications were chosen since these are common in the Netherlands. In line with Okely et al. [31], the four items could be answered on a 5-point Likert scale. The second psychosocial factor “*Affective response*” was separated into negative affective impact and positive affective impact. *“Negative affective impact”* was measured using a subscale of the CardiacTM questionnaire, adapted for pregnancy (e.g. “Because of my heart incident I worry more about my health” was reframed into “Because of my pregnancy I worry more about my health”). The subscale consisted of nine questions that could be answered on 7-point Likert scale. *“Positive affective impact”* was measured using a validated Dutch version of the Positive And Negative Affect Scale (PANAS) [34], similar to the approach of Pesonen et al. [35] in their study among pregnant women. On a 5-point Likert scale, participants could indicate the extent to which they experienced 10 positive emotions during their pregnancy. The third psychosocial factor *“Changed self-concept”* was assessed using subscales from the CardiacTM questionnaire, adjusted for pregnancy (e.g. “The way I see my lifestyle in the future has changed because of my heart incident” was reframed into “The way I see my lifestyle in the future has changed because of my pregnancy”). In addition, similar to the study of Okely [31], we included an item that asks for the importance of the mother or ‘mother to be’ identity (a new social identity that may arise during pregnancy) because change in identity may affect health behavior change [36, 37]. The final scale consisted of eight questions were scored on a 7-point Likert scale. The fourth psychosocial factor *“Social support”* was measured using the Dutch validated version of the 12-item Multidimensional Scale of Perceived Social Support (MSPSS) questionnaire [38, 39], which has strong factorial and construct validity in pregnant women [40]. A question on partner support was added based on literature displaying the importance of partner support during pregnancy [41, 42]. This question was formulated based on earlier research using the TM framework, adapted for the topic of lifestyle (e.g. “My partner supports me in adhering to a lifestyle appropriate for a healthy pregnancy”) [31]. The resulting 13 items regarding social support were scored on a 7-point Likert scale. Measurements of all factors resulted in an average score, with higher scores representing higher levels of the measured factor.

#### Intentions to change behavior

*Intentions to change behavior* were assessed using the validated Cardiac Lifestyle Change Intention (LCI) scale [32], originally aimed to assess whether an acute cardiac event has led to subsequent intention to change lifestyle. We adapted the original statements of the scale for the event of pregnancy (e.g. “My heart attack has made me realize that a healthy lifestyle is important to me” was reframed into “Pregnancy has made me realize that a healthy lifestyle is important to me”). The final 11 statements, scored on a 7-point Likert scale, will be referred to as the Pregnancy Lifestyle Change Intention (PLCI) score.

#### Health behaviors

Six health behaviors that are relevant during pregnancy were measured (i.e. dietary quality, folic acid supplementation, vitamin D supplementation, smoking, use of alcohol and physical activity). Dietary quality was assessed retrospectively using an adapted short version of the Dutch Healthy Diet Food Frequency Questionnaire (DHD-FFQ) [43]. Usual daily intake of vegetables, fruits, grain products and fish was measured based on Dutch nutritional guidelines and specific requirements for pregnant women [44, 45]. From the dietary intake data, a dietary quality score was constructed. For each of the four components, a minimum score of 0 and a maximum score of 10 was obtained. These component scores were summed, resulting in an overall dietary quality score ranging from 0 to 40 [46]. For supplementation of folic acid and vitamin D, a dichotomous score (Y/N) for current use was reported. Smoking, use of alcohol and physical activity were measured using questions based on a study on prenatal care [47, 48]. Women were asked if they had smoked or used alcohol in the past 14 days (Y/N) and if they ever smoked or used alcohol before. If so, they were asked about the reason for quitting (‘pregnancy’ or ‘planning pregnancy’ or ‘different’). To assess sufficiency of physical activity (PA), women were asked how many times per week they exercised for at least 30 min consecutively. A dichotomous score (Y/N) was generated based on the Dutch recommendations for PA of 5 times 30 min of moderate physical activity per week [49]. Women were asked their opinion on their level of PA and if unsatisfied a reason for low level of PA was asked.

### Analyses

For the descriptive analyses we used frequencies and percentages for categorical variables and mean (SD) for continuous variables when they were normally distributed. Participants with a mean pregnancy lifestyle change intention score (PLCI) score of $\ge$ 5 (slightly agree on a 7-point Likert scale) were denoted as participants with increased intentions to change health behavior due to pregnancy. This approach is in line with previous studies [50, 51]. An independent-samples t-test was performed to assess differences between the 2 groups with high and low PCLI scores in Tables 1 and 2. The associations between the TM factors (independent variables) and the PLCI score (dependent variable) were analyzed by univariate and multivariate linear regression. Assumptions for the linear regression analyses (linearity, independence of residuals, homoscedasticity, and normal distribution of residuals) were checked first. We then assessed the univariate association between each individual TM factor and the PCLI score. In multivariate model 1, we assessed the association between each individual TM factor and the PCLI score, and additionally corrected each association for the identified confounders. In multivariate model 2, we included all TM factor in one model and again corrected for the identified confounders. Regression coefficients indicating associations with the dependent variable were expressed as ß with a 95% CI and a p-value was presented. For the multivariate regression models R-square was calculated. The association between ‘intentions to change behavior’ and dietary quality was analyzed by linear regression, after checking for assumptions regarding these analyses. The associations between ‘intention to change’ and vitamin D supplementation and physical activity were analyzed by logistic regression. Assumptions for logistic regression analyses were checked (linearity, independence of observations, multicollinearity, outliers), the Box-Tidwell procedure showed no linear relationship. Therefore, the dichotomized PLCI score was used, divided in lower ($<$ 5) PLCI scores and higher $(\ge$ 5) PLCI scores [52]. Results were expressed as odds ratio (OR) with a 95% confidence interval (CI) and the p-value for the crude and adjusted models. Folic acid supplementation, smoking and alcohol consumption were not included in the model because of low numbers in the different response categories.

**Table 1:** Sociodemographic and pregnancy-related characteristics

Characteristic	Total (*N* = 343)		PLCI* Score < 5 (*N* = 150)		PLCI* Score ≥ 5 (*N* = 193)		P-value
	Mean (SD)		Mean (SD)		Mean (SD)		
**Age**	30.5 (4.14)		30.0 (4.04)		30.8 (4.20)		0,761
	**N (%)**		**N (%)**		**N (%)**		
**Relationship status**							0,419
Married/in relationship	333 (97.1)		145 (96.7)		188 (97.4)		
Single	10 (2.9)		5 (3.3)		5 (2.6)		
**Employment status**							0,598
Employed	307 (89.5)		135 (90)		172 (89.1)		
Not employed/on benefits	36 (10.5)		15 (10)		21 (10.9)		
**Employment status partner**							0,032
Employed	325 (97.6)		143 (98.6)		182 (96.8)		
Not employed/on benefits	8 (2.4)		2 (1.4)		6 (3.2)		
**Education** ^**a**^							0,004
High	258 (75)		107 (71.3)		151 (78.2)		
Low	85 (25)		43 (28.7)		42 (21.8)		
**Ethnicity**							0,172
No migration background	280 (81.7)		120 (80)		160 (82.9)		
Migration background	63 (18.3)		30 (20)		33 (17.1)		
**Primiparous**							< 0,001
Yes	217 (63.3)		84 (56)		133 (69.3)		
No	126 (36.7)		66 (44)		59 (30.7)		
**Pre-pregnancy BMI**							< 0,001
< 30 Kg/m2	296 (88.1)		121 (81.8)		175 (93.1)		
≥ 30 Kg/m2	40 (11.9)		27 (18.2)		13 (6.9)		
**Gestational age**							0,644
≤ 12 Weeks	72 (21)		27 (18.1)		45 (23.3)		
13–24 Weeks	111 (32.4)		48 (32.2)		63 (32.6)		
> 24 Weeks	159 (46.6)		74 (49.7)		85 (44)		
**Planned pregnancy**							< 0,001
Yes	313 (91.3)		131 (87.3)		182 (94.3)		
No	30 (8.7)		19 (12.7)		11 (5.7)		
**Time to conception**							0,034
≤ 1 Year	299 (87.2)		134 (89.3)		165 (85.5)		
> 1 Year	44 (12.8)		16 (10.7)		28 (14.5)		
**Natural conception**							0,751
Yes	310 (90.4)		136 (90.7)		174 (90.2)		
No	33 (9.6)		14 (9.3)		19 (9.8)		
**Complications** **current pregnancy** ^**b**^							0,066
Yes	123 (35.9)		58 (38.7)		65 (33.7)		
No	220 (64.1)		92 (61.3)		128 (66.3)		
**Complications** **previous pregnancy** ^**b**^							0,009
Yes	54 (43.7)		28 (42.4)		26 (44)		
No	71 (56.3)		38 (57.8)		33 (56)		
**Received/inquired info on lifestyle during pregnancy** ^**c**^							< 0,001
Yes	289 (84.3)		119 (79.3)		170 (88.1)		
No	54 (15.7)		31 (20.7)		23 (11.9)		

*Pregnancy Lifestyle Change Intention score (scale 1–7)

a: Education ‘low’ consists of; low education (elementary school, “VMBO-basis/kader/gemengd”, “LBO”) and middle education (“MAVO”, “MBO”, “VMBO-theoretisch”, “MTS”, “BOL”, “BBL”, “INAS”). Education ‘high’ consists of “HAVO”, “VWO”, “HTS”, “HBO” and “WO”

b: Complications: Gestation diabetes, hyperemesis gravidarum, severe pelvic instability, high blood pressure, preeclampsia or other complications

c: Searched or received information from friends, family or healthcare professionals such as general practitioners, gynecologists, midwifes, fertility specialists, nurses, dieticians, yoga teachers or doulas

**Table 2:** Lifestyle characteristics

Characteristic	Total (*N* = 343)	PLCI* Score < 5 (*N* = 150)	PLCI* Score ≥ 5 (*N* = 193)	P-value
	Mean (SD)	Mean (SD)	Mean (SD)	
**Dietary quality score (0–40)**	23.14 (5.99)	22.23 (5.83)	23.83 (6.04)	0,938
	**N (%)**	**N (%)**	**N (%)**	
**Vitamin D supplementation** ^**a**^				< 0.001
Yes	296 (86.3)	123 (78)	173 (89.7)	
No	47 (13.7)	27 (22)	20 (10.3)	
**Folic acid supplementation** ^**b**^				0.001
Yes	341 (99.4)	148 (98.7)	193 (100)	
No	2 (0.6)	2 (1.3)	0 (0)	
**Smoking (past 14 days)**				0.005
Yes	9 (2.6)	6 (4)	3 (1.5)	
No	334 (97.4)	144 (96)	190 (88.5)	
**Smoking (ever)** ^**c**^	109 (31.8)	50 (33.3)	59 (30.5)	0.907
Quit when trying to conceive	25 (23)	10 (20)	15 (25.4)	
Quit when pregnant	29 (26.6)	16 (32)	13 (22)	
Quit for other reasons	55 (50.4)	24 (48)	31 (52.6)	
**Alcohol intake (past 14 days)**				0.108
Yes	3 (0.9)	2 (1.3)	1 (0.5)	
No	340 (99.1)	148 (98.7)	192 (99.5)	
**Alcohol intake (ever)** ^**c**^	300 (87.5)	130 (86.7)	170 (88.1)	0.260
Quit when trying to conceive	100 (33.3)	36 (27.7)	64 (37.6)	
Quit when pregnant	166 (55.3)	80 (61.5)	86 (50.6)	
Quit for other reasons	34 (11.4)	14 (10.8)	20 (11.8)	
**Physical activity sufficient**				< 0.001
Yes	94 (27.3)	25 (16.7)	69 (35.7)	
No	249 (72.7)	125 (83.3)	124 (64.3)	

* Pregnancy lifestyle change intention score (scale 1–7)

a: Refers to current supplementation of vitamin D started either before conception, before 4 weeks of pregnancy or later

b: Refers to folic acid supplementation earlier in pregnancy, started either before conception, before 4 weeks of pregnancy or later

c: Refers to former health behavior, answering the question: “Have you ever smoked (or used alcohol) before?”

Each model was adjusted for potential confounders identified based on literature (age, ethnicity, employment status, level of education, relationship status, gestational age, primiparous, current or previous pregnancy complications, planned pregnancy, conception with medical assistance, duration to conceive, pre-pregnancy BMI and information about lifestyle and pregnancy). These covariates are explained in Additional file 2. A subset of confounders was selected for inclusion in each model. In line with established practices for confounder selection [53], we checked whether inclusion of each potential confounder changed the ß by more than 10%. Potential confounders were tested per health behavior. Selected confounders were added to the univariate and multivariate models in a stepwise manner, ensuring a minimum of 15 cases per variable as previously recommended [54]. All analyses were performed using SPSS (version 25; IBM; Armonk, NY).

## Results

### Sample characteristics

For the final sample of 343 women sociodemographic and pregnancy-related characteristics of the study population are depicted in Table 1, lifestyle characteristics are presented in Table 2. These tables show the characteristics of the complete sample and the results separated between women reporting lower ($<$ 5) PLCI scores, versus women reporting higher $(\ge$ 5) PLCI scores. The final sample consisted of women averaging 30.5 years of age, with the majority of those women having completed higher education (75%), being employed (89.5%), and being married or in a relationship (97.1%). Most women were pregnant with their first child (63.3%), most pregnancies were planned (91.3%) and the majority of women were in their third trimester at the time of data collection (46.6%).

Table 2 shows that the vast majority of women reported taking folic acid (99.4%) and vitamin D supplements (86.3%). A minority of women reported to attain the recommended level of physical activity (27.3%). Very few women reported cigarette smoking or alcohol use in the last 14 days (2.6% and 0.9%).

### Prevalence of increased intentions to change health behavior due to pregnancy

Results demonstrate that 56% of the pregnant women scored an averaged mean score of ≥ 5 on the PLCI score, whereas 44% of the sample scored an averaged mean score of < 5 on the PLCI score.

### Associations between TM factors and intentions to change health behavior due to pregnancy

Table 3 depicts the median pregnancy lifestyle change intention (PLCI) score and the median scores for the different TM factors. It shows for instance that our respondents experience a lot of social support (6.3 on a scale of 1–7) and perceive a low risk of their lifestyle on their pregnancy (2 on a scale of 1–7). In Table 4, multivariate model 2 demonstrated that respectively both higher scores for positive and negative affective impact were associated with higher intentions to change health behavior (positive β = 0.28; CI = 0.21–0.48, negative β = 0.12; CI = 0.00-0.15). A higher changed self-concept score was associated with higher intentions to change health behavior (β = 0.21; CI = 0.11–0.31). Higher perceived lifestyle risk was associated with lower intentions to change health behavior (β=-0.29; CI = 0.31 − 0.13). No association was found for the factors perceived risk pregnancy and social support in multivariate model 2.

**Table 3:** Median (IQR) and range of the PLCI and TM factors

Characteristic	Median (IQR)	Range	N
**Dependent variable**			
Pregnancy lifestyle change intention score	5.1 (4.5–5.5)	1–7	343
**Independent variables (TM factors)**			
Affective impact positive	3.6 (3.2-4.0)	1–5	343
Affective impact negative	3.3 (2.4–4.3)	1–7	343
Perceived risk pregnancy	2.0 (1.8–2.5)	1–5	343
Perceived risk lifestyle	2.0 (1.5-3.0)	1–7	343
Changed self-concept	4.8 (4.1–5.2)	1–7	334
Social support	6.3 (5.7–6.8)	1–7	342

IQR: Interquartile range

PLCI score: Pregnancy lifestyle change intention score

TM: Teachable moment

**Table 4:** Univariate and Multivariate Regression Analyses between TM factors and the PLCI

Independent variables (TM factors)	Univariate analyses		Multivariate model 1		Multivariate model 2 (R^2^ = 0.36)	
	β	95% CI	β	95% CI	β	95% CI
Affective impact positive	0.36***	0.32, -0.57	0.36***	0.32, 0.57	0.28***	0.21, 0.48
Affective impact negative	-0.07	-0.11, 0.02	-0.01	-0.07, 0.07	0.12*	0.00, 0.15
Perceived risk pregnancy	-0.16*	-0.29, -0.30	-0.14**	-0.31, -0.04	-0.01	-0.15, 0.12
Perceived risk lifestyle	-0.33***	-0.33, -0.19	-0.31***	-0.31, -0.15	-0.29***	-0.31, -0.13
Changed self-concept	0.30***	0.19, 0.40	0.32***	0.22, 0.41	0.21***	0.11, 0.31
Social support	0.24***	0.12, 0.31	0.18**	0.06, 0.27	0.04	-0.06, 0.14

Note. Multivariate model 1; multivariate regression analyses with 1 independent TM factor per model, corrected for: age, education, ethnicity, pre-pregnancy BMI, planned pregnancy, primiparous, time to conception, natural conception, complications current pregnancy and complications pervious pregnancy

Multivariate model 2; multivariate regression analyses with 6 independent TM factors in one model, corrected for: age, education, ethnicity, pre-pregnancy BMI, planned pregnancy, primiparous, time to conception, natural conception, complications current pregnancy and complications previous pregnancy

*p < 0.05 **p < 0.01 ***p < 0.001

TM: Teachable moment

### Association between intentions to change health behavior and current self-reported health behaviors

The multivariate analyses presented in Table 5 show that a higher intention to change health behaviors was significantly associated with higher self-reported dietary quality scores (β = 0.11; CI = 0.82–1.64). Furthermore, a higher intention to change health behaviors was significantly associated with higher PA levels (OR = 2.88; CI = 1.66-5.00). The multivariate logistic analysis established no significant association between intention to change and Vitamin D supplementation.

**Table 5:** Univariate and multivariate regression analyses between PLCI and self-reported health behaviors

Characteristic	Univariate analyses		Multivariate analyses	
**Dietary quality** ^**a**^	**β**	**95% CI**	**Β**	**95% CI**
PLCI score†	0.14**	0.30–1.90	0.11*	0.82–1.64
**Vitamin D supplementation** ^**b**^	**OR**	**CI**	**OR**	**CI**
PLCI score††	1.90*	1.02–3.54	1.47	0.77–2.83
**Physical activity** ^**c**^	**OR**	**CI**	**OR**	**CI**
PLCI score††	2.78***	1.65–4.68	2.88***	1.66-5.00

a: Dietary quality. Multivariate linear analysis corrected for: age, education, planned pregnancy, primiparous, time to conception, complications current pregnancy and information inquired or received

b: Vitamin D supplementation. Multivariate logistic analysis corrected for: pre-pregnancy BMI and primiparous

c: Physical activity. Multivariate logistic regression analysis corrected for: gestational age, pre-pregnancy BMI and primiparous

**†** Pregnancy lifestyle change intention score, applied as a continuous variable, range 1–7

**††** Pregnancy lifestyle change intention score, applied as a dichotomous variable, high score (≥ 5) versus low score (< 5)

*p < 0.05 **p < 0.01 ***p < 0.001

## Discussion

This study aimed at obtain more insight in whether a pregnancy evokes a potential TM and which psycho-social factors related to teachable moments are associated with an increased intention to change health behaviors due to pregnancy. We also intended to investigate the association between increased intentions to change behavior and current health behaviors. We found that the majority of pregnant women in our sample (56%) reported increased pregnancy lifestyle change intentions as a result of their pregnancy, indicating a possible occurrence of a TM. Higher intentions to change health behaviors due to pregnancy were mainly associated with increased levels of positive affective impact and change in self-concept and to a lesser extent by increased levels of negative affective impact. Women reporting higher levels of perceived risk, showed lower levels of intentions to change health behavior. Finally, we found that higher intentions to change health behaviors were associated with two improved self-reported current behaviors; healthier diet and increased physical activity.

Our finding that the majority of women experienced higher intentions to change health behavior due to pregnancy is in line with previous research. Bookari et al. [55] discovered high levels of motivation to change nutritional behavior during pregnancy among 70% of the participants. Lindqvist [28] found that most pregnant women wanted to increase their physical activity, improve their dietary habits and lose weight. Qualitative studies found women reporting high levels of motivation to adopt a healthy lifestyle [56] and discovered that a TM was provoked predominantly in women who conceived with fertility treatment, as the treatment raised their levels of affective response, risk perception and change in self-concept [29]. We found no meaningful difference in intentions to change health behavior between women conceiving with or without fertility treatment.

We found that women who experience higher affective impact as a result of their pregnancy, were more likely to show favorable behavior changes. These findings confirm the TM theory [10] and are in line with research on affect in relation to health behavior [57]. In our study, women experiencing positive affect (i.e. feeling for instance strong, enthusiastic and proud about their pregnancy) were more inclined to change their health behavior compared to women experiencing negative affect (i.e. feeling worry, sadness and fear about their pregnancy and their own and their baby’s health). Comparable to our findings, several studies show that positive affect is linked to favorable health behavior change [58, 59]. This could be explained by recent qualitative studies showing that positive affect raised motivation, self-confidence and self-efficacy, which prompted beneficial health behavior change during pregnancy [60, 61]. Our findings on negative affect are confirmed by a qualitative study explaining that negative emotions (e.g. boredom, stress, frustration) increased women’s motivation to make positive health behavior changes [61]. Positive health behavior changes following negative affect - such as fear and worry about the health of the fetus - can be explained by an increased motivation to eliminate health risks [10, 57]. Too much fear can have adverse effects, causing health behavior to remain unchanged or even to deteriorate [10].

Our findings show that women perceiving higher lifestyle-related risk, were less inclined to change their health behavior. We did not find an association with pregnancy-related risk. The findings on perceived risk lifestyle may be explained by participants having made beneficial changes to their health behaviors before they participated in our study, and therefore, perceive less risk towards their health or pregnancy. This could be confirmed by our results demonstrating almost 50% of our participants quit smoking and nearly 90% abstained from drinking due to pregnancy or trying to conceive. When people modify health behavior, they also lower their risk perceptions [62]. The low scores reported for both subscales of perceived risk imply that women in our study viewed themselves and their unborn babies not very susceptible to this risk. An additional potential explanation is that risk perception and actual risk are two distinct concepts. Previous studies indicate that risk perception and actual risk during or after pregnancy are not always linked and women may underestimate their risks [63, 64]. According to the Health Belief Model - describing that an individual’s perceived susceptibility influences the decision to take health-related action - this may reduce the likelihood of increased intentions to change health behavior [65].

When comparing our findings to existing studies, we found contrasting results in a study investigating the TM model after a gestational diabetes diagnosis [31]. The increased levels of risk perception related to higher adherence to treatment of gestational diabetes could be explained by the fact that a disease diagnosis leads to higher levels of risk perception and urgency compared to improving health behavior during pregnancy. Rockliffe et al. [61] confirms our findings and explains that perceived risk sometimes acts as a barrier to change behavior (e.g. Some women would refrain from physical activity, believing it could hurt the baby) underlining the importance of knowledge and understanding about risks to prevent low risk perception. The need for clear messages is highlighted in a study that reported hinder due to confusing messages provided by professionals [61, 66]. Risk perceptions are complex, highly individualized and are based on more than medical diagnoses (e.g. trust in healthcare). Therefore, risk perceptions should ideally be discussed during antenatal consultations [67, 68].

Self-concept is defined as the perception of oneself or one’s position in life [69]. Our findings demonstrate that the more women reported that pregnancy changed their self-concept, the more they wanted to improve their health behaviors. This association is in line with recent qualitative studies showing that during pregnancy, women were keen to fulfil what they perceived to be their new role or identity of ‘good mother’, predominantly driven by the health of the baby and by social expectations [60, 61]. Our findings can be further explained by connection between the new ‘mother-identity’ and health behavior change. Identity plays an important role in health behavior change, people are inclined to act in line with their identity [37]. Important life-events can shift people’s identity, causing people to re-evaluate their current health behavior and compare whether these behaviors are in line with their new identity [70]. In this way, a shift in identity or self-concept prompts health behavior change [37].

Social support did not significantly affect intentions to change health behavior. When comparing our findings, we found overall results for social support during pregnancy to be ambiguous. Studies either reported small [42, 71, 72] or no associations [73]. An explanation for this ambiguity could be that social support - including partner support - was found to act as both a barrier and a facilitator for health behavior change [61, 71]. For instance, partner support could be to quit smoking, while a partner could hinder by continuing to smoke inside. Contrasting to our findings, Okely et al. [31] did find a significant association between social support and adherence to treatment. A possible explanation could be that a gestational diabetes diagnosis compared to a normal pregnancy, leads to more participation from loved-ones, whether or not stimulated by healthcare professionals.

Our final aim was to explore the relation between intentions to change health behaviors and current self-reported health behaviors. We found that women more apt to change health behavior reported a healthier diet and higher levels of physical activity during pregnancy compared to women reporting lower intentions to change. Furthermore, the majority of women in our study reported to have spontaneously quit smoking and drinking when either trying to conceive or pregnant. These findings are in line with previous research [74, 75]. McBride [10] showed that increased intentions to change, combined with self-efficacy and skill acquisition, may increase the probability that an individual will engage in health protective behavior. Not all intentions to change behavior are translated into actual behaviors. This is referred to as the intention-behavior gap [76]. Indeed, although significant, our effect-sizes were relatively small. It further highlights the need that other factors related to behavior change (e.g. practical skills and barriers) to support lifestyle change during teachable moments should be further investigated.

### Strengths and limitations

Our study had several strengths. To our knowledge, this is the first quantitative study to evaluate pregnancy itself as a TM, employing a theoretical framework designed by Mcbride et al. [10]. Also, our large sample size provided us with enough power to perform the analyses. Moreover, this study linked factors in this framework to actual health behaviors.

Nonetheless, several limitations should be considered. First, health behaviors were self-reported, which may differ from actual behavior [77]. Second, due to social pressure to behave like ‘a good mother’, socially desirable answers may have been reported [61, 78]. Third, data collection took place during the COVID pandemic, which might have influenced women’s experience of pregnancy related to lifestyle behavior intentions, as confirmed in a recent study showing that the COVID pandemic evoked a TM regarding lifestyle change in cardiovascular disease patients [79]. Fourth, the cross-sectional design limits the possibility to make causal inferences about mechanisms involved in TM. Fifth, this study’s external validity and thereby generalizability of the results could be affected in several ways. Participants recruited via online forums might have a higher-than-average interest in healthy lifestyle and thus might be more inclined to fill out the questionnaire. We therefore encourage scholars to further study TM’s in other populations such as those living in vulnerable situations or with different cultural backgrounds, because the decisions and choices related to lifestyle need to be considered within the context in which they are made. Recent work by Locke et al. [78] highlights the need for a wider consideration of contextual factors that are shaping health behaviors.

### Implications and future research

Our findings on risk perception, affect and changed self-concept provide us with several possible ramifications for prenatal care. First, it is important to carefully monitor pregnant women’s mental state, to connect negative affect to risk-reducing health behaviors and to offer help when negative emotions overtake [10, 57]. Second, focus towards the utilization of positive affect during pregnancy should be intensified, as positive emotions such as feeling strong, enthusiastic and proud demonstrated potential to increase health behavior change intentions [58, 59]. Third, focus on the connection between health behavior change and a women’s changed identity and her new role of mother should be integrated in prenatal care [37, 70]. Fourth, our results on perceived lifestyle risk underscore the importance of providing clear and timely information [61, 67]. More knowledge about risk perception and behavior change during pregnancy is needed as our study shows potential undesired effects of a higher risk perception. The importance of social support should be acknowledged, a tailored approach and more research are recommended [61, 71]. Behavior change during pregnancy isn’t merely an individual process and must be viewed in the wider context of society and the contexts of people’s lives [78].

Pregnancy is different from other potential TM’s [80] as it’s a temporary unique physiological process, requiring women to make decisions for another individual and changing multiple health behaviors [81]. Further research about TM’s during pregnancy are needed, preferably based on theoretical models adapted for pregnancy [60, 61]. Extended follow up data is necessary to gain knowledge about the timing as well as intensity of TM’s across subgroups. Cohen et al. [13] found that TM’s can be provoked in moments of interaction with healthcare professionals. Educating healthcare professionals to be aware of this possibility could accelerate positive health behavior change [13, 14]. Research on how to impact and provoke TM’s throughout pregnancy during moments of contact with healthcare professionals is essential for actual adoption of acquired knowledge [13]. Finally, information about pregnancy-related health behavior change and its underlying associated factors could be applied in interventions to improve health behaviors of pregnant women [61]. In the process of designing and implementing preventive interventions using a TM however, it is crucial to acknowledge the phenomenon that preventive interventions that aim to improve overall health might widen existing inequalities within the population, as those in more vulnerable situations may respond and benefit less [82, 83].

## Conclusion

In summary, the majority of the women in our sample experience increased intentions to change health behavior due to pregnancy, indicating a teachable moment was provoked. Increased intentions to change health behavior due to pregnancy were associated to experiencing affective impact - predominantly positive affect -, a change in a woman’s self-concept and risk perception. In turn, intentions to change health behavior were associated to two current self-reported health behaviors: diet and physical activity. These results point out that pregnancy could become an important teachable window, providing opportunities for health professionals and governments for lifestyle advice. Our findings suggest that communication about pregnancy-related health behaviors should be linked to a women’s change in self-concept, affective impact (mainly positive affect) and risk perception. Effectively using teachable moments during pregnancy has the potential to facilitate positive health behavior changes with less efforts. As pregnancy is an event occurring in the majority of women, advances in health behavior during pregnancy may lead to improved health of current and next generations.

### Electronic supplementary material

Below is the link to the electronic supplementary material.

Supplementary Material 1

Supplementary Material 2

## Data Availability

The datasets used and/or analyzed during the current study are available from the corresponding author on reasonable request.
