# Supplementary material for: A cross-sectional analysis of factors associated with the teachable moment concept and health behaviors during pregnancy
Source: BMC Pregnancy Childbirth. 2024 Feb 20;24:147. doi: 10.1186/s12884-024-06348-8 (PMC10880280; doi:10.1186/s12884-024-06348-8)
Supplement: Supplementary file 1 — Supplementary Material 1 [file 12884_2024_6348_MOESM1_ESM.docx]

Additional file 1

Questionnaire – General Information

1. **What is your age?** Fill out: XX years.
2. **What is your marital status?**

- Married or registered partnership, living together
- Married or registered partnership, not living together
- In a relationship, living together
- In a relationship, not living together
- Single

1. **Do you currently have a paid job or are you in social welfare?**

- Yes, a paid job for more than 24 hours per week
- Yes, a paid job for less than 24 hours per week
- Yes, social welfare
- No

1. **If you have a partner, does your partner currently have a paid job or social welfare?**

- Yes, a paid job for more than 24 hours per week
- Yes, a paid job for less than 24 hours per week
- Yes, social welfare
- No
- Not applicable, I don’t have a partner.

1. **In which country were you born?**

- The Netherlands
- Suriname
- Netherlands Antilles / Aruba
- Türkiye/Turkey
- Maroc
- In (another) European county, North America, Australia, New Zealand, Indonesia or Japan.
- Other

**5a. In which country was your father born?**

- The Netherlands
- Suriname
- Netherlands Antilles / Aruba
- Türkiye/Turkey
- Maroc
- In (another) European county, North America, Australia, New Zealand, Indonesia or Japan.
- Other

**5b. In which country was your mother born?**

- The Netherlands
- Suriname
- Netherlands Antilles / Aruba
- Türkiye/Turkey
- Maroc
- In (another) European county, North America, Australia, New Zealand, Indonesia or Japan.
- Other

**6. What is the highest level of education you have completed?**

(Completed with a diploma or sufficient certificate)

- No education or only primary education (elementary school, special primary education)
- Lower or preparatory vocational education (such as VMBO basic/intermediate/mixed, LBO)
- Secondary general education (such as MAVO, MBO-1, VMBO, theoretical program)
- Secondary vocational education and apprenticeships (such as MBO-2/3/4, MTS, BOL, BBL, INAS)
- Higher general secondary education (such as HAVO, VWO)
- Higher vocational education (such as HTS, HBO)
- University education
- Other

Questionnaire - Pregnancy

The following questions are about your pregnancy and possible previous pregnancies

1. **How many weeks pregnant are you?** Complete: XX
2. **Was this a planned pregnancy?**

- Yes
- No -> continue with question 5

1. **How long did it take to conceive?**

- I was pregnant within 6 months
- I was pregnant between 6 and 12 months
- I was pregnant after 12 months

1. **Did you conceive in a natural way or did you have medical help?** *(Medical help through, for example, IUI, IVF, ICSI or by taking hormones)*

- I conceived naturally
- I conceived through medical help

1. **Did you suffer from pregnancy-related complaints during this pregnancy?**

*(multiple answers possible)*

- No
- Severe pelvic instability
- Severe nausea (hyperemesis gravidarum)
- Gestational diabetes
- High blood pressure
- Preeclampsia
- Other

1. **What was your approximate weight in the month before you were pregnant?** Fill out in kilograms. XXX
2. **What is your height in centimeters?** Fill out in centimeters XXX
3. **Have you given birth before?**

- Yes
- No 🡪 continue with question 11

1. **Have you suffered from complications during your previous pregnancies?**

*(for example: Severe pelvic instability, severe nausea, gestational diabetes, high blood pressure or preeclampsia)*

- Yes
- No

1. **Have you suffered from complications during previous deliveries?**

- Yes
- No

1. **Have you received or searched for information regarding a healthy life style during pregnancy?**

- Yes
- No

1. **What person or what source did you receive information from about a healthy lifestyle during pregnancy?**

*(multiple answers possible)*

- I searched for information on the internet myself
- from family and friends
- from the general practitioner
- from the midwife
- from the gynecologist/OBGYN
- from the fertility doctor
- from the nurse
- during pregnancy yoga
- from the dietitian
- from a doula
- Other

Questionnaire – Pregnancy Lifestyle Change Intention (PLCI)
Adapted from Cardiac Lifestyle Change Intention scale(1)

7-Point Likert Scale:

Strongly Disagree – Disagree – Slightly Disagree – Neutral – Slightly Agree – Agree – Strongly Agree

**Factor 1: Influence of Pregnancy on Lifestyle**

- 1. I am working hard to improve my lifestyle.
  2. I have made positive adjustments to my lifestyle.
  3. Due to my pregnancy, I feel a greater need for a healthy lifestyle.
  4. Because of my pregnancy, I allow myself more time to live healthily.
  5. My pregnancy has made me realize that a healthy lifestyle is important to me.
  6. Due to my pregnancy, I have started living more healthily.
  7. I see the period around my pregnancy as a fresh start.

**Factor 2: Intentions for a Healthy Lifestyle**

- 1. I am always motivated to live healthily.
  2. In my opinion, my lifestyle is fine as it is.
  3. In most situations, I live a healthy lifestyle.
  4. I am easily tempted to live unhealthily. (Reversed item)

Questionnaire - TM factors (McBride et al., 2003)

Adapted from the Cardiac Teachable Moment scale(1) and the Dutch version of the Positive And Negative Affect Scale (PANAS)(2)

**Factor 1: Positive affect related to pregnancy.** (I PANAS-SF positive)

5-point scale (To a great extent – Hardly or not at all)

When I think about my pregnancy, to what extent did I feel...

1. Interested
2. Excited
3. Strong
4. Enthusiastic
5. Proud
6. Alert
7. Inspired
8. Determined
9. Attentive
10. Active

**Factor 2: Negative Affect Related to Pregnancy**

7-Point Likert Scale:

Strongly Disagree – Disagree – Slightly Disagree – Neutral – Slightly Agree – Agree – Strongly Agree

1. When I start worrying about my pregnancy, I can't stop worrying.
2. When I start worrying about my health, I can't stop worrying.
3. When I start worrying about the health of my baby, I can't stop worrying.
4. Due to my pregnancy, I worry more about my health.
5. The worries I have about my pregnancy and/or my baby affect my emotions.
6. The worries I have about my pregnancy and/or my baby affect my daily life.
7. Since I became pregnant, I have felt sad more often.
8. Since I became pregnant, I have felt anxious more often.
9. Since I became pregnant, I have felt down more often.

**Factor 3: Risk perception regarding pregnancy**

5 Answer Options (1-5) from 'Not at all or hardly' to 'Very high'

**Factor 3.1: Risk perception regarding the baby**

1. I assess the risk of my baby having a low birth weight as...

2. I assess the risk of my baby being born prematurely as...

**Factor 3.2: Risk perception regarding yourself**

3. I assess the risk of developing gestational diabetes myself during this pregnancy as...

4. I assess the risk of developing high blood pressure or (pre-)eclampsia myself during this pregnancy as...

**Factor 4: Risk perception regarding lifestyle**

7-Point Likert Scale:

Strongly Disagree – Disagree – Slightly Disagree – Neutral – Slightly Agree – Agree – Strongly Agree

1. There is a good chance that I will develop lifestyle-related diseases at some point in my life.
2. I estimate the likelihood of developing lifestyle-related diseases in the next ten years as high.
3. If I continue with my current lifestyle, I expect to have health problems for sure.
4. If I continue with my current lifestyle, I expect health problems for my baby.
5. I believe that my risk of lifestyle-related diseases is higher than other people of my age and gender.

**Factor 5: Identification with pregnant women**

7-Point Likert Scale:

Strongly Disagree – Disagree – Slightly Disagree – Neutral – Slightly Agree – Agree – Strongly Agree

1. I do not feel a connection with other pregnant women. (Reversed)
2. Due to my pregnancy, I feel more connected to other pregnant women.
3. I feel a kinship with other pregnant women.

**Factor 6: Identity and lifestyle**

7-Point Likert Scale:

Strongly Disagree – Disagree – Slightly Disagree – Neutral – Slightly Agree – Agree – Strongly Agree

1. Due to my pregnancy, I feel worse about myself when I don't exercise.
2. Due to my pregnancy, I feel worse about myself when I don't take time for relaxation.
3. Due to my pregnancy, I feel worse about myself when I eat unhealthily.
4. Due to my pregnancy, I feel worse about myself when I smoke.
5. Due to my pregnancy, I feel worse about myself when I consume alcohol.

**Factor 7: Change in social role**

7-Point Likert Scale:

Strongly Disagree – Disagree – Slightly Disagree – Neutral – Slightly Agree – Agree – Strongly Agree

1. My role as a spouse/romantic partner has become more important to me due to my pregnancy.
2. Due to my pregnancy, my role as a future mother has become important to me.
3. Due to my pregnancy, I realize better how important I am to my loved ones.
4. Due to my pregnancy, I realize more how valuable my life actually is.
5. Due to my pregnancy, I appreciate myself more.

Questionnaire – Social Support (Multidimensional Scale of Perceived Social Support (MSPSS))(3)

7-Point Likert Scale

Below are a number of statements about emotional support one can receive from their surroundings. Choose the answer that best applies. There are no right or wrong answers; your own impression is the only thing that matters.

1. There is a special person who is around when I am in need. 1 2 3 4 5 6 7

2. There is a special person with whom I can share my joys and sorrows. 1 2 3 4 5 6 7

3. My family really tries to help me. 1 2 3 4 5 6 7

4. I get the emotional help and support I need from my family. 1 2 3 4 5 6 7

5. I have a special person who is a real source of comfort to me. 1 2 3 4 5 6 7

6. My friends really try to help me. 1 2 3 4 5 6 7

7. I can count on my friends when things go wrong. 1 2 3 4 5 6 7

8. I can talk about my problems with my family. 1 2 3 4 5 6 7

9. I have friends with whom I can share my joys and sorrows. 1 2 3 4 5 6 7

10. There is a special person in my life who cares about my feelings. 1 2 3 4 5 6 7

11. My family is willing to help me make decisions. 1 2 3 4 5 6 7

12. I can talk about my problems with my friends. 1 2 3 4 5 6 7

**Support from partner**

7-Point Likert Scale

13. My partner supports me in adapting a life style that fits with a healthy pregnancy.

Questionnaire: Dutch Healthy Diet Food Frequency Questionnaire (DHD-FFQ) (4).

The following questions are about your nutrition and (possible) intake of nutritional supplements.

| 1. | **On average, how many slices of bread or rolls do you eat per day?** | | |  |
| --- | --- | --- | --- | --- |
|  | 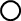 | I don’t eat bread or rolls (**Continue with question 2)** | |  |
|  | 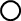 | 1-2 slices/pieces | |  |
|  | 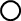 | 3 slices/pieces | |  |
|  | 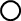 | 4 slices/pieces | |  |
|  | 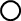 | 5 slices/pieces | |  |
|  | 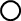 | 6 or more slices/pieces | |  |
|  | 1a. | **Which type of bread or rolls do you usually eat?** | |  |
|  | 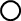 | | Whole-wheat, brown, or multi-grain | |
|  | 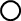 | | White | |
|  | 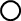 | | Both whole-wheat, brown, or multi-grain as, well as white | |
|  | 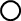 | | other / I don’t know | |

| 2. | **On average how many days per week do you eat rice, pasta, couscous, or other grain products?** | |
| --- | --- | --- |
|  | 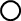 | I don’t eat rice, pasta, couscous or other grain products **(⇾ continue with question 3)** |
|  | 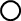 | Less than 1 day per week |
|  | 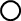 | 1-2 days per week |
|  | 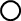 | 3-4 days per week |
|  | 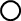 | 5-6 days per week |
|  | 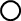 | every day |
|  | 2a. | **How many serving spoons (±50 grams) do you eat on average on such day?** |
|  | 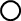 | 1 serving spoon |
|  | 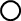 | 2 serving spoons |
|  | 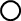 | 3 serving spoons |
|  | 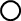 | 4 serving spoons |
|  | 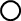 | 5 serving spoons |
|  | 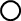 | 6 or more serving spoons |
|  | 2b. | **What kind of rice, pasta, couscous or other grain products do you usually eat?** |
|  | 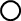 | whole-wheat or brown |
|  | 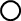 | white |
|  | 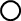 | both whole-wheat, brown and white |
|  | 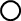 | other / I don’t know |

| 3. | | **On average how many days per week do you eat cooked or stir-fried vegetables?** | | | | | | |
| --- | --- | --- | --- | --- | --- | --- | --- | --- |
|  | | 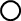 | | | I don’t eat cooked or stir-fried vegetables (**continue with questions 4)** | | | |
|  | | 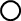 | | | Less than 1 day per week | | | |
|  | | 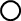 | | | 1-2 days per week | | | |
|  | | 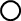 | | | 3-4 days per week | | | |
|  | | 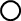 | | | 5-6 days per week | | | |
|  | | 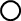 | | | every day | | | |
|  | | 3a. | | | **How many serving spoons (±50 grams) do you eat on average on such day?** | | | |
|  | | 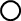 | | | | 1 serving spoon | |  |
|  | | 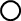 | | | | 2 serving spoons | |  |
|  | | 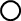 | | | | 3 serving spoons | |  |
|  | | 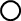 | | | | 4 serving spoons | |  |
|  | | 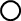 | | | | 5 serving spoons | |  |
|  | | 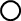 | | | | 6 or more serving spoons | |  |
| 4. | | **On average how many days per week do you eat vegetable salad?** | | | | | | |
|  | | *Vegetable salad include for example lettuce, carrot, cucumber, and other kinds of uncooked vegetables.* | | | | | | |
|  | | 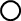 | | | I don’t eat vegetable salad (**⇾ continue with question 5)** | | | |
|  | | 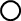 | | | Less than 1 day per week | | | |
|  | | 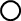 | | | 1-2 days per week | | | |
|  | | 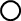 | | | 3-4 days per week | | | |
|  | | 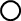 | | | 5-6 days per week | | | |
|  | | 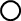 | | | every day | | | |
|  | |  | | |  | | | |
|  | 4a. | | **How many servings (±50 gram) do you eat on average on such day?** | | | | | |
|  | | 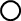 | Less than 1 serving | | | |  |  |
|  | | 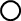 | 1 serving | | | |  |  |
|  | | 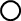 | 2 servings | | | |  |  |
|  | | 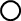 | 3 servings | | | |  |  |
|  | | 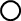 | 4 servings | | | |  |  |
|  | | 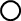 | 5 or more servings | | | |  |  |

| 5 | **On average how many days per week do you eat a serving fish?** | | | |
| --- | --- | --- | --- | --- |
|  | *A serving fish includes for example herring, a piece of salmon, fried fish, a trout, etc.* | | | |
|  | 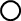 | I don’t eat fish **(⇾ continue with question 6)** | | |
|  | 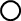 | Less than 1 day per week | | |
|  | 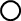 | 1 day per week | | |
|  | 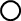 | 2 days per week | | |
|  | 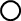 | 3 days per week or more | | |
|  | 5a. | **What type of fish do you usualy eat?** | | |
|  | 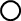 | | Fatty fish (for example salmon, mackerel, herring) |  |
|  | 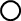 | | Lean fish (for example tuna, cod, fried fish, tilapia, or other white fish) |  |
|  | 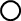 | | Both fatty and lean fish |  |
|  | 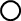 | | other / I don’t know |  |

| 6 | **On average how many days per week do you eat fruit?** | | | |
| --- | --- | --- | --- | --- |
|  | 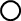 | | I don’t eat fruit **(⇾ Continue with question 7)** | |
|  | 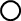 | | Less than 1 day per week | |
|  | 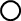 | | 1-2 days per week | |
|  | 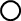 | | 3-4 days per week | |
|  | 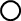 | | 5-6 days per week | |
|  | 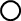 | | every day | |
|  | 6a. | | **How many servings do you eat on average on such day?** | |
|  | *A serving is for example 1 apple, 1 banana, 2 tangerines, 1 small bowl of strawberries, 1 slice of melon.* | | | |
|  | 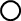 | Less than 1 serving | |  |
|  | 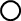 | 1 serving | |  |
|  | 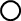 | Sometimes 1, sometimes 2 servings | |  |
|  | 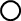 | 2 or more servings | |  |

**7. Have you taken folic acid?**

- Yes, I started (over) 4 weeks before I was pregnant
- Yes, I started between the 2^nd^ and 4^th^ week of pregnancy (Usually this is the moment when tested positive on pregnancy)
- Yes, I started after the 4^th^ week of pregnancy
- No

**8. Do you take vitamin D?**

- Yes, I started (over) 4 weeks before I was pregnant
- Yes, I started between the 2^nd^ and 4^th^ week of pregnancy (Usually this is the moment when tested positive on pregnancy)
- Yes, I started after the 4^th^ week of pregnancy
- No

Questionnaire: Smoking and alcohol

The following questions are on the topic of smoking and use of alcohol.

**9. Did you smoke in the last 14 days?**

- No 🡪 *9a*
- Yes, but no more than 5 cigarettes per week
- Yes, but no more than 5 cigarettes per day
- Yes, but more than 5 cigarettes per day

**9a. Have you ever smoked?**

- No, I have never smoked
- Yes, but I stopped smoking when I learned I was pregnant
- Yes, but I stopped smoking because of my pregnancy wish
- Yes, but I stopped smoking for another reason than having children or being pregnant.

**10. Did you drink alcohol in the last 14 days?**

- No *-> 10a*
- Yes, sometimes, but no more than 2 drinks per week
- Yes, but no more than 4 drinks per week
- Yes, 5 drinks or more per week

**10a. Have you ever consumed alcohol?**

- No, I have never consumed alcohol
- Yes, but I stopped drinking when I learned I was pregnant
- Yes, but I stopped drinking because of my pregnancy wish
- Yes, but I stopped drinking for another reason than having children or being pregnant.

Questionnaire: Physical activity

The following questions are on the topic of physical activity

**11. Last week, how many times have you exercised for at least 30 minutes?**

*(Exercise means your heart rate increases, for example: walking, cycling, or working out)*

- Not once
- 1 time per week
- 2 times per week
- 3 times per week
- 4 times per week
- 5 or more times per week

**12. Are you content with your frequency of exercising?**

**My frequency of exercise is:**

- - - More than sufficient (continue with question. xx)
    - Sufficient (continue with question. xx)
    - Moderate
    - Insufficient

**13. You indicated that your frequency of exercise is moderate or insufficient. Can you indicate the reason?**

*(Multiple answers are allowed)*

- Due to physical complaints related to pregnancy
- Due to physical complaints, but unrelated to pregnancy
- I don’t have time
- Other

References

1. Brust M, Gebhardt WA, van der Voorde NAE, Numans ME, Kiefte-de Jong JC. The development and validation of scales to measure the presence of a teachable moment following a cardiovascular disease event. Prev Med Rep. 2022;28:101876.

2. Engelen U, Peuter SD, Victoir A, Diest IV, Van den Bergh O. Verdere validering van de Positive and Negative Affect Schedule (PANAS) en vergelijking van twee Nederlandstalige versies. gedrag en gezondheid. 2006;34(2):61-70.

3. Zimet GD, Powell SS, Farley GK, Werkman S, Berkoff KA. Psychometric characteristics of the Multidimensional Scale of Perceived Social Support. J Pers Assess. 1990;55(3-4):610-7.

4. van der Velde LA, Nyns CJ, Engel MD, Neter JE, van der Meer IM, Numans ME, et al. Exploring food insecurity and obesity in Dutch disadvantaged neighborhoods: a cross-sectional mediation analysis. BMC Public Health. 2020;20(1):569.
