# Supplementary material for: A cross-sectional analysis of factors associated with the teachable moment concept and health behaviors during pregnancy
Source: BMC Pregnancy Childbirth. 2024 Feb 20;24:147. doi: 10.1186/s12884-024-06348-8 (PMC10880280; doi:10.1186/s12884-024-06348-8)
Supplement: Supplementary file 2 — Supplementary Material 2 [file 12884_2024_6348_MOESM2_ESM.docx]

**Additional file 2**

**Methods, explanation covariate selection**

Several covariates were identified based on literature and collected in the questionnaire. McBride emphasizes the importance to consider individuals’ dispositional factors, cultural characteristics and predisposing factors such as age (1) (years), ethnicity (1, 2) (migration background Y/N) as well as employment status(2) and level of education, (2-5) because these factors may impact the factors of the conceptual framework and intentions to change. Employment status was evaluated for both women and partner as either ‘employed’ or ‘unemployed/on benefits’. Level of education was measured as low education (none, elementary or vocational education), middle education (higher general and secondary vocational education) or high education (higher professional and scientific education). Data showed 3 cases in category ‘low’, therefore the categories ‘low’ and ‘middle’ were combined. Level of education was categorized into ‘low’ and ‘high’ education. Relationship status (‘married/in relationship’ or ‘single’) has an impact on the level of social support. Additionally, relationship status was found to impact intentions to change health behavior (6). Gestational age (measured in weeks, evaluated in first, second or third trimester) is relevant because it often relates to physical symptoms such as nausea, fatigue or changes in energy and mobility (7). These symptoms can affect both the factors of conceptual framework as well as intentions to change health behavior. The experience of having a baby influences risk perception, affective impact, change in self-concept and intentions to change. Therefore, women were asked if this was their first baby (Y/N) (8). Current or previous pregnancy complications (gestation diabetes, hyperemesis gravidarum, severe pelvic instability, high blood pressure, preeclampsia or other complications) may influence the framework’s factors and intention to change (4, 9). For example, if a woman suffered severe nausea in a previous pregnancy this may change their level of affect and risk perception considerably. Current or previous complications were analyzed as a dichotomous variable (Y/N). Whether a pregnancy was planned (Y/N) was found to have an effect on health behaviors during pregnancy (10). Research showed that women who conceived with medical assistance (Y/N) were more likely to experience increased intentions to change as a result of an increase in the psychosocial factors (11). Also, women’s health behaviors improved when it took longer to conceive, therefore the duration of the preconception period was measured ($\leq$ 1 year or $>$ 1 year) (12). Pre-pregnancy BMI was linked to intentions to change health behavior and was measured in Kg/m2 (13, 14). Finally, knowledge was found to influence risk perception in pregnant women (15). Therefore, women were asked if they inquired or received information concerning lifestyle and pregnancy (Y/N), and if so, who they received information from (general practitioners, gynecologists, midwifes, fertility specialists, nurses, dieticians, yoga teachers or Doula’s).

**References**

1. McBride CM, Emmons KM, Lipkus IM. Understanding the potential of teachable moments: the case of smoking cessation. Health Educ Res. 2003;18(2):156-70.

2. Hillier SE, Olander EK. Women's dietary changes before and during pregnancy: A systematic review. Midwifery. 2017;49:19-31.

3. Crone MR, Luurssen-Masurel N, Bruinsma-van Zwicht BS, van Lith JMM, Rijnders MEB. Pregnant women at increased risk of adverse perinatal outcomes: A combination of less healthy behaviors and adverse psychosocial and socio-economic circumstances. Preventive Medicine. 2019;127:105817.

4. Okely J, Mason C, Collier A, Dunnachie N, Swanson V. Diagnosis of gestational diabetes: a 'teachable moment'. Diabetic Medicine. 2019;36(2):184-94.

5. Heslehurst N, Hayes L, Jones D, Newham J, Olajide J, McLeman L, et al. The effectiveness of smoking cessation, alcohol reduction, diet and physical activity interventions in changing behaviours during pregnancy: A systematic review of systematic reviews. PLoS One. 2020;15(5):e0232774.

6. Jonsdottir SS, Thome M, Steingrimsdottir T, Lydsdottir LB, Sigurdsson JF, Olafsdottir H, et al. Partner relationship, social support and perinatal distress among pregnant Icelandic women. Women and Birth. 2017;30(1):e46-e55.

7. Olander EK, Darwin ZJ, Atkinson L, Smith DM, Gardner B. Beyond the 'teachable moment' - A conceptual analysis of women's perinatal behaviour change. Women Birth. 2016;29(3):e67-71.

8. Bastian LA, Pathiraja VC, Krause K, Namenek Brouwer RJ, Swamy GK, Lovelady CA, et al. Multiparity is associated with high motivation to change diet among overweight and obese postpartum women. Womens Health Issues. 2010;20(2):133-8.

9. Harrison AL, Taylor NF, Shields N, Frawley HC. Attitudes, barriers and enablers to physical activity in pregnant women: a systematic review. Journal of Physiotherapy. 2018;64(1):24-32.

10. Yanikkerem E, Ay S, Piro N. Planned and unplanned pregnancy: effects on health practice and depression during pregnancy. Journal of Obstetrics and Gynaecology Research. 2013;39(1):180-7.

11. Atkinson L, Shaw RL, French DP. Is pregnancy a teachable moment for diet and physical activity behaviour change? An interpretative phenomenological analysis of the experiences of women during their first pregnancy. British Journal of Health Psychology. 2016;21(4):842-58.

12. Wise LA, Wesselink AK, Hatch EE, Weuve J, Murray EJ, Wang TR, et al. Changes in behavior with increasing pregnancy attempt time: a prospective cohort study. Epidemiology. 2020;31(5):659-67.

13. Lindqvist M, Lindkvist M, Eurenius E, Persson M, Mogren I. Change of lifestyle habits - Motivation and ability reported by pregnant women in northern Sweden. Sexual and Reproductive Healthcare. 2017;13:83-90.

14. Merkx A, Ausems M, Budé L, de Vries R, Nieuwenhuijze MJ. Weight gain in healthy pregnant women in relation to pre-pregnancy BMI, diet and physical activity. Midwifery. 2015;31(7):693-701.

15. Bayrampour H, Heaman M, Duncan KA, Tough S. Predictors of perception of pregnancy risk among nulliparous women. Journal of Obstetric, Gynecologic & Neonatal Nursing. 2013;42(4):416-27.
